# Comparative Analysis of Context-Dependent Mutagenesis in Humans and Fruit Flies

**DOI:** 10.1155/2013/173616

**Published:** 2013-08-05

**Authors:** Sofya A. Medvedeva, Alexander Y. Panchin, Andrey V. Alexeevski, Sergey A. Spirin, Yuri V. Panchin

**Affiliations:** ^1^Department of Bioengineering and Bioinformatics, Moscow State University, Vorbyevy Gory 1-73, Moscow 119992, Russia; ^2^Institute for Information Transmission Problems, Russian Academy of Sciences, Bolshoi Karetny Pereulok 19-1, Moscow 127994, Russia; ^3^Department of Mathematical Methods in Biology, Belozersky Institute, Moscow State University, Vorbyevy Gory 1-40, Moscow 119991, Russia; ^4^Department of Mathematics, Scientific-Research Institute for System Studies, Russian Academy of Sciences, Nakhimovskii Prospekt 36-1, Moscow 117218, Russia

## Abstract

In general, mutation frequencies are context-dependent: specific adjacent nucleotides may influence the probability to observe a specific type of mutation in a genome. Recently, several hypermutable motifs were identified in the human genome. Namely, there is an increased frequency of T>C mutations in the second position of the words ATTG and ATAG and an increased frequency of A>C mutations in the first position of the word ACAA. Previous studies have also shown that there is a remarkable difference between the mutagenesis of humans and drosophila. While C>T mutations are overrepresented in the CG context in humans (and other vertebrates), this mutation regularity is not observed in *Drosophila melanogaster*. Such differences in the observed regularities of mutagenesis between representatives of different taxa might reflect differences in the mechanisms involved in mutagenesis. We performed a systematical comparison of mutation regularities within 2–4 bp contexts in *Homo sapiens* and *Drosophila melanogaster* and found that the aforementioned contexts are not hypermutable in fruit flies. It seems that most mutation contexts affect mutation rates in a similar manner in *H. sapiens* and *D. melanogaster*; however, several important exceptions are noted and discussed.

## 1. Introduction

The average rates of point mutations in multicellular eukaryotic genomes are usually between 10^−7^ and 10^−10^ mutations per nucleotide per generation [1, 2]. However, the rates of point mutations may be dramatically altered by their genomic context. In some cases, this context-dependent change in mutation frequency can be attributed to known molecular mechanisms involved in mutagenesis. For example, the increased frequency of C>T mutations in the word CG in humans (and other vertebrates) is attributed to the methylation of cytosines by context-specific DNA methyltransferases [3]. This mutation regularity is absent in *D. melanogaster* [4], in which cytosine methylation occurs, but appears to be restricted to early embryonic development and is not specific to cytosines followed by guanines [5]. Many other examples of context-dependent mutagenesis have been reported [4, 6–9].

Recently, an increased rate of T>C mutations in the second position of the words ATTG and ATAG and an increased rate of A>C mutations in the first position of the ACAA word were reported in the human genome [10]. This was achieved by calculating the values called “minimal contrast” and “mutation bias” for 2–4 bp mutation contexts to evaluate if the addition of specific nucleotides to the 5′ or 3′ end of 1–3 bp words increases the probability of observing certain mutations in fixed positions. Mutation bias indicates the total excess (or deficiency) of mutations within a given context. Minimal contrast indicates the excess (or deficiency) of mutations within a given context that cannot be explained by the excess (or deficiency) of mutations in one of its subcontexts.

*H. sapiens* and *D. melanogaster *are perspective model organisms for this kind of studies because of the vast amount of data on genetic variation that is available for them. The goal of our study was to compare the mutation regularities of *H. sapiens* and *D. melanogaster* in terms of “minimal contrast” and “mutation bias.”

## 2. Methods

We searched for single nucleotide variable positions in intergenic sequences of 37 individual *D. melanogaster *genomes (multiple alignments obtained from http://genome.ucsc.edu/ [11]). *Drosophila sechellia *(droSec1, Oct. 2005) and *Drosophila erecta* (droEre2, Feb. 2006) genomic sequences were used as outgroups to reconstruct the ancestral states for the variable positions. *D. melanogaster* genome (dm3, Apr. 2006) was used as the reference.

### 2.1. Mutation Data

We assume that a mutation with a known direction within a known context has occurred in a specific position of the *D. melanogaster *genome if the following conditions are met.

1. *D. sechellia* and *D. erecta *genomes have the same nucleotide aligned to this position (this nucleotide will be referred to as the “ancestral nucleotide”).
2. Among the 37* D. melanogaster *genomes, some contain the ancestral nucleotide in this position, while some other genomes contain a different nucleotide.
3. Only 2 genetic variants are present in this position for the 37* D. melanogaster *genomes.
4. The 3 bp upstream and downstream positions from these positions in the multiple alignment do not contain any substitutions or gaps.

Mutation bias and minimal contrasts for* D. melanogaster *were calculated for 2–4 bp mutation contexts using the methods described in [10]. Mutation bias, contrasts, and other data for *H. sapiens* were taken directly from [10].

### 2.2. Mutation Context and Subcontext

We denote the mutation context of mutation mut in position pos of the word W as {mut | pos, W}. For example, {C>T ∣1, CG} represents a C>T mutation in the first position of the word CG. Mutation context  {mut | pos′, W′} is called a subcontext of the context {mut | pos, W} if W′ is a subword of W, and any mutation mut occurring in position pos of the word W is at the same time a mutation occurring in position pos′ of the word W′. For example, {C>T ∣1, CG}  is a subcontext of {C >T ∣2, ACG}.

### 2.3. Contrast

For each pair of context {mut | pos, W} and its subcontext {mut | pos′, W′}, the value of contrast is given by the formula

(1) Contrast({mut ∣ pos,W},{mut ∣ pos',W'}) =P{mut ∣ pos,W}P{mut ∣ pos',W'}.

Here, *P*_{mut∣pos,W}_ and *P*_{mut∣pos*'*,W*'*}_ are the conditional probabilities of observing mutation mut in the position pos of the word W and position pos′ of word W′, respectively, in a given dataset. Although these probabilities cannot be explicitly calculated without assumptions of the general probability of mutation per nucleotide in the genome, their ratio can be estimated by the following formula:

(2) P{mut ∣ pos,W}P{mut ∣ pos',W'}=N{mut ∣ pos,W}/PWN{mut ∣ pos',W'}/PW′  .

Here, *P*_W_ and *P*_W′_ are the observed frequencies of words W and W′, respectively, among all words of the same length. *N*_{mut∣pos,W}_ and *N*_{mut∣pos*'*,W*'*}_ are the observed numbers of mutation mut in position pos of wordWand position pos′ of the word W′, respectively. 

The ratio *P*_W_/*P*_W′_ estimates the probability for W′ to be extended to W. This ratio coincides with the expected ratio *N*_{mut∣pos,W}_/*N*_{mut∣pos′,W′}_ under the hypothesis that mutations rates are the same in the context {mut | pos, W} and its subcontext {mut | pos′, W′}. Therefore, if Contrast ({mut | pos, W},{mut | pos′, W′}) is greater than 1, it indicates an increased mutation rate in the context {mut | pos, W} compared with the subcontext {mut | pos′, W′}; while if Contrast ({mut | pos, W},{mut | pos′, W′}) is less than 1, it indicates a decreased mutation rate.

### 2.4. Minimal Contrast

For a given context {mut | pos, W}, let us consider all of its subcontexts {mut | pos′, W′}. The minimal contrast is the value MC = Contrast ({mut | pos, W},{mut | pos′, W′}) such that the absolute difference *|*MC − 1*|* is the lowest among all subcontexts {mut | pos′, W′}. We did not study discontigvous contexts such as CNG and CNNG.

### 2.5. Mutation Bias

For any context {mut | pos, W}, there exists only one subcontext {mut | pos′, W′} such that the length of W′ is equal to 1 (i.e., W′ is the one-letter word consisting of the mutated letter). The mutation bias is the contrast of the given context and this subcontext.

### 2.6. Word Frequencies

We used two measures of *D. melanogaster *word frequencies. The first measure was obtained using complete aligned sequences of 37 *D. melanogaster*, the *D. sechellia*, and *D. erecta* genomes. For the second measure, we used conserved regions in which the ancestral nucleotide matches at least one of the *D. melanogaster *genetic variants, and no gaps or unread sequences are present in the multiple alignment. Word frequencies from the conserved regions were used for calculating mutation biases and contrasts.

## 3. Results and Discussion

The nucleotide composition of complete alignments and conserved regions (see Section 2) of *D. melanogaster *were similar (Table 1). We decided to use word frequencies within conserved regions of *D. melanogaster *for calculations of contrast and mutation bias.

**Figure 1:**
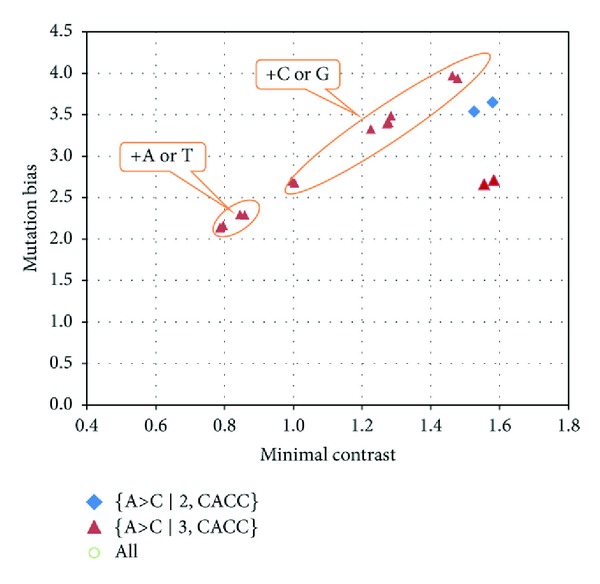
Mutation bias and minimal contrasts of mutation contexts in* D. melanogaster*. Each dot represents a mutation context. Triangles represent the {A>C ∣3, CCA} (as well as complementary contexts) and contexts that had this context as a subcontext. Most dots are in pairs because complementary contexts have similar mutation bias and minimal contrast values.

Previous studies have shown that the representation of mutation data on a plot of mutation bias versus minimal contrast is useful for identifying important mutation contexts [10]. Mutation bias and minimal contrasts of mutation contexts in *D. melanogaster* are shown in Figure 1. The {A>C ∣2, CACC} and {A>C ∣3, CCA} mutation contexts have the highest minimal contrast values in *D. melanogaster. *Interestingly, the addition of C or G nucleotides to either end of the word CCA increases mutation bias of the A>C mutation, while the addition of A or T nucleotides to these words decreases mutation bias.

As shown in Table 2, mutation patterns differ between *D. melanogaster *and *H. sapiens* at the single nucleotide scale: *D. melanogaster* has a lower transition/transversion ratio. Moreover, the G>T (C>A) transversion in *D. melanogaster *comprises a much larger fraction of mutations than the A>G (T>C) transition, which is consistent with previous findings [4].

One of the mechanisms by which G>T (C>A) transversions occur is through the formation of 8-Oxoguanine [12] caused by reactive oxygen species [13] or ultraviolet irradiation [14]. In eukaryotes, the damaged DNA is repaired with the help of DNA glycosylase OGG1. This enzyme removes the 8-oxoguanine, forming a DNA apurinic-apyrimidinic site, which is then recognized by other proteins of the DNA repair system. If further reparation does not occur, the apurinic-apyrimidinic site will be complemented with an adenine nucleotide during DNA replication, resulting in a C>A mutation. Another protein with DNA glycosylase activity for 8-hydroxyguanine, called dOgg1, was also described in *D. melanogaster *[15].

Another factor that might be responsible for increased G>T (C>A) transversion rates in *D. melanogaster *is aflatoxin B1. Aflatoxin B1 is known to induce base substitutions in DNA [16, 17], especially G>T (C>A) transversions. It is a product of a fungus from the *Aspergillus *genus, which grows on fruits and grains in a humid climate; thus, it is quite possible that *D. melanogaster *is exposed to this toxin.

**Figure 2:**
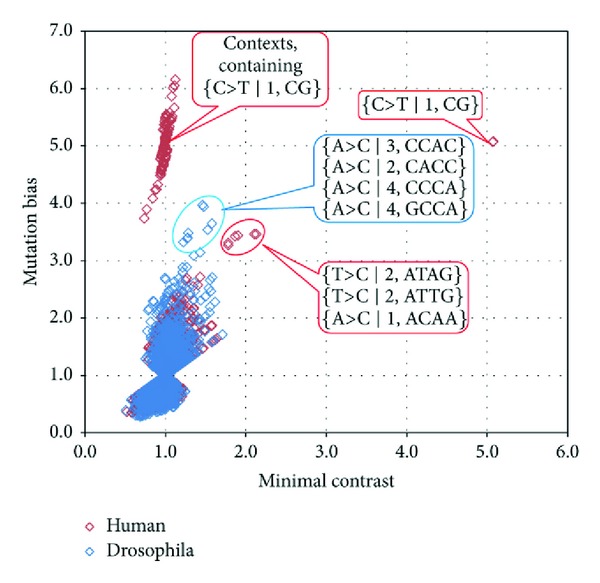
Mutation bias and minimal contrast for *D. melanogaster *and *H. sapien*s. Each dot represents a mutation context (blue in *D. melanogaster*, red in *H. sapiens). *Dots are overlapping and are usually in pairs because complementary contexts have similar mutation bias and minimal contrast values.

*D. melanogaster *and *H. sapien*s mutageneses are also strikingly different for several 2–4 bp contexts, as shown in Figure 2. The {C>T ∣1, CG}, {T>C ∣2, ATTG}, {T>C ∣2, ATAG}, and {A>C ∣1, ACAA} mutation contexts appear to have excessive mutation frequencies in *H. sapiens* but not in *D. melanogaster. *Interestingly, the CAATT sequence (contains the ATTG word on the reverse strand) appears to be a mutation hotspot for the human DNA polymerase eta [18]. Also, the CCAAT (contains the ATTG word on reverse strand) motif is a known target site for enhancer-binding proteins [19]. The increased number of ATTG>ACTG mutations might be partially due to selection against enhancers sequences in nontranscribed regions of the genome.

**Figure 3:**
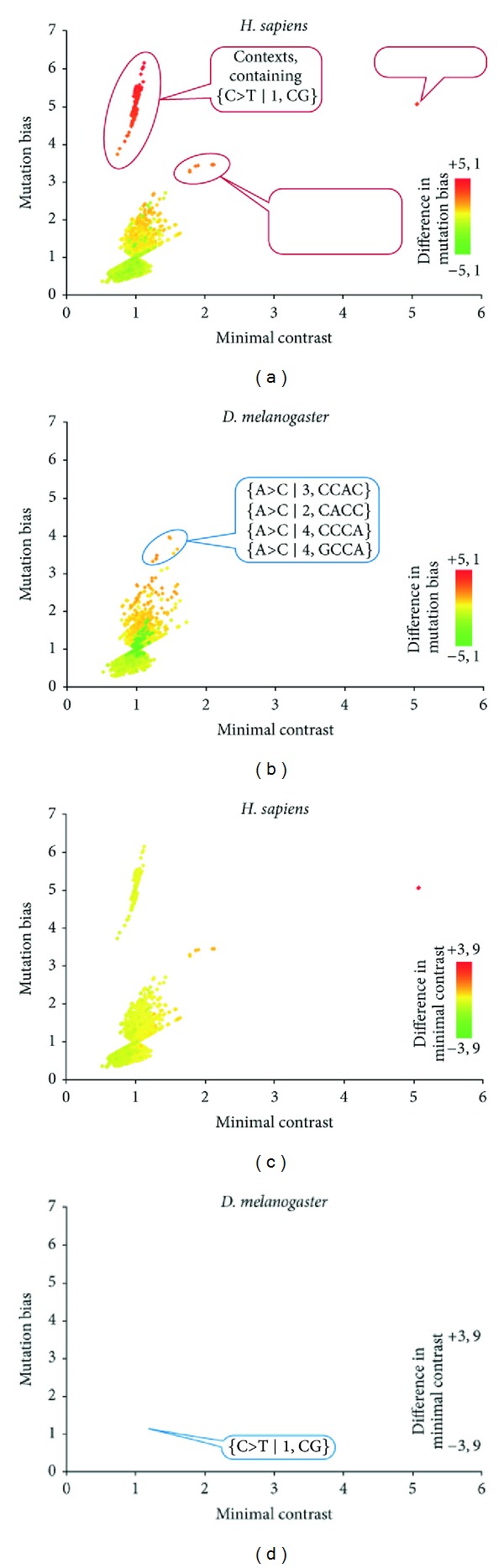
The difference between *H. sapiens* and *D. melanogaster* mutation bias ((a) and (b)) and minimal contrast ((c) and (d)) for 2–4 bp mutation contexts. Each dot represents a mutation context. The *X* axis represents the contexts minimal contrast values, and the *Y* axis represents the contexts mutation bias. The minimal contrast and mutation bias values are given for *H. sapiens* ((a) and (c)) and for *D. melanogaster *((b) and (d)), and the color scheme indicates the difference between minimal contrasts. Thus, red dots on (a) and (c) represent contexts that are hypermutable in humans comparing to drosophila, while green dots represent contexts that are hypermutable in *D. melanogaster* comparing to *H. sapiens*. This scheme is reversed for (b) and (d).

On the other hand, several mutation contexts seem to have increased mutation bias in *D. melanogaster. *The differences between different mutation contexts in *D. melanogaster* and *H. sapiens* are shown in more detail in Figure 3.

In a previous study, we compared the over- and underrepresentation of 1–7 bp nucleotide words in the genomes of 139 complete eukaryotic genomes, including *H. sapiens* and *D. melanogaster* [20].  Table 3 contains a part of this comparison for several words in *H. sapiens* and *D. melanogaster *related to the previously discussed mutation contexts. The word CG has a strong underrepresentation in *H. sapiens* (by 76.37% from the expected genomic frequency) while in *D. melanogaster* it is only slightly underrepresented (by 5.93% from the expected genomic frequency). The derived word TG is overrepresented by 20.1% and by 10.67% in* H. sapiens *and *D. melanogaster,* respectively. The {C>T ∣1, CG} mutation context seems to be the only example of a mutation context that has remarkably affected the genomic word composition in *H. sapiens *compared to* D. melanogaster*. The absence of such effects for words related to other mutation contexts might be due to us not taking into account the rates of other mutations in these words or mutations that produce these words.

## 4. Conclusions

The regularities of mutagenesis are different in *D. melanogaster *and *H. sapiens*. However, these differences may be attributed to a rather small number of mutation contexts that behave in a different manner in these two species. First, there is an increased frequency of G>T (C>A) transversions in *D. melanogaster. *Several possible molecular mechanisms for this have been proposed. Second, there is an increased frequency of C>T mutation in the word CG in *H. sapiens*. This is probably explained by the fact that human germline methylation is abundant and CpG specific, while *D. melanogaster* is not. Third, there is an increased frequency of T>C mutations in the second position of the words ATTG and ATAG and an increased frequency of A>C mutations in the first position of the ACAA word in *H. sapiens* but not in *D. melanogaster*. And finally, there is an increased A>C mutations rate in {A>C ∣2, CACC} and {A>C ∣3, CCA} mutation contexts in *D. melanogaster* but not in* H. sapiens*.

## Figures and Tables

**Table 1:** Comparison of nucleotide composition of complete alignments and conserved regions of *D. melanogaster*.

Nucleotide	Nucleotide fraction within all positions	Nucleotide fraction within conserved positions	Difference, %
a	0.2979	0.2901	2.6
t	0.2978	0.2899	2.7
c	0.2022	0.2100	−3.9
g	0.2021	0.2100	−3.9

**Table 2:** Comparison of single nucleotide mutations in *D. melanogaster *and *H. sapien*s. Transitions are italic, while transversions are bold.

*D. melanogaster *		*H. sapiens *	
Mutation	Fraction	Mutation	Fraction
**A>C**	**0,044**	**A>T**	**0,031**
**T>G**	**0,047**	**T>A**	**0,031**
**C>G**	**0,047**	**A>C**	**0,037**
**G>C**	**0,048**	**T>G**	**0,038**
**A>T**	**0,057**	**C>G**	**0,051**
**T>A**	**0,058**	**G>C**	**0,051**
*A>G *	*0,063 *	**G>T**	**0,058**
*T>C *	*0,064 *	**C>A**	**0,058**
**G>T**	**0,118**	*T>C *	*0,118 *
**C>A**	**0,121**	*A>G *	*0,118 *
*C>T *	*0,166 *	*C>T *	*0,204 *
*G>A *	*0,167 *	*G>A *	*0,204 *
**Transversions**	**0,540**	**Transversions**	**0,355**
*Transitions *	*0,460 *	*Transitions *	*0,645 *

**Table 3:** Over- and underrepresentation of genomic frequencies for several words in *H. sapiens* and *D. melanogaster*. Data is taken from a previous study [20] supplementary table (available at http://mouse.genebee.msu.ru/words/Supple3(contrast_k).xls). The numbers represent the value *C* = [(Obs (W) – Exp (W))/Exp (W)] · 100%, where Obs (W) is the observed word frequency and Exp (W) is the expected word frequency (based on the frequencies of all of its subwords).

	Genomic word over- and underrepresentation in	
	*H. sapiens*	*D. melanogaster*
Words containing a mutation context with increased mutation bias in *H. Sapiens *		
CG	−76.37%	−5.93%
ATAG	−0.79%	4.38%
ATTG	−7.07%	−2.35%
ACAA	1.62%	3.75%
Words derived from mutation contexts with increased mutation bias in *H. Sapiens *		
TG	20.10%	10.67%
ACAG	1.51%	−4.94%
ACTG	−2.07%	−0.46%
CCAA	−6.17%	−1.61%
Words containing mutation contexts with increased mutation bias in *D. melanogaster *		
CCAC	0.19%	1.52%
CACC	1.18%	−4.24%
CCCA	5.63%	0.09%
GCCA	−2.77%	3.63%
ACC	2.28%	−2.39%
CCA	14.82%	9.90%
Words derived from mutation contexts with increased mutation bias in *D. melanogaster *		
CCCC	−5.10%	2.19%
GCCC	1.66%	−1.41%
CCC	−12.66%	−7.78%
